# Annual variations of monsoon and drought detected by GPS: A case study in Yunnan, China

**DOI:** 10.1038/s41598-017-06095-1

**Published:** 2017-07-19

**Authors:** Weiping Jiang, Peng Yuan, Hua Chen, Jianqing Cai, Zhao Li, Nengfang Chao, Nico Sneeuw

**Affiliations:** 1 GNSS Research Center, Wuhan University, Wuhan, 430079 China; 2 School of Geodesy and Geomatics, Wuhan University, Wuhan, 430079 China; 3 Institute of Geodesy, University of Stuttgart, Stuttgart, 70174 Germany; 4 Faculté des Sciences, de la Technologie et de la Communication, University of Luxembourg, 6, rue Richard Coudenhove-Kalergi, L-1359 Luxembourg City, Luxembourg; 5 MOE Key Laboratory of Fundamental Physical Quantities Measurement, School of Physics, Huazhong University of Science and Technology, Wuhan, 430074 China

## Abstract

The Global Positioning System (GPS) records monsoonal precipitable water vapor (PWV) and vertical crustal displacement (VCD) due to hydrological loading, and can thus be applied jointly to diagnose meteorological and hydrological droughts. We have analyzed the PWV and VCD observations during 2007.0–2015.0 at 26 continuous GPS stations located in Yunnan province, China. We also obtained equivalent water height (EWH) derived from the Gravity Recovery And Climate Experiment (GRACE) and precipitation at these stations with the same period. Then, we quantified the annual variations of PWV, precipitation, EWH and VCD and provided empirical relationships between them. We found that GPS-derived PWV and VCD (positive means downward movement) are in phase with precipitation and GRACE-derived EWH, respectively. The annual signals of VCD and PWV show linearly correlated amplitudes and a two-month phase lag. Furthermore, the results indicate that PWV and VCD anomalies can also be used to explore drought, such as the heavy drought during winter/spring 2010. Our analysis results verify the capability of GPS to monitor monsoon variations and drought in Yunnan and show that a more comprehensive understanding of the characteristics of regional monsoon and drought can be achieved by integrating GPS-derived PWV and VCD with precipitation and GRACE-derived EWH.

## Introduction

The Global Positioning System (GPS) observes precipitable water vapor (PWV) and vertical crustal displacement (VCD) due to hydrological loading, and thus has the potential for applications to monitor monsoon variations and meteorological and hydrological droughts^1^. Moreover, terrestrial water storage (TWS) derived from the Gravity Recovery And Climate Experiment (GRACE) and conventional meteorological observations (e.g. precipitation data) are also used for drought monitoring^2–4^. Therefore, integrating GPS, GRACE and meteorological observations to diagnose drought has been an important issue in space geodesy and relevant research fields.

Continuous GPS height time series have been used to estimate the crustal deformation due to water mass loading in regions with monsoon climate, such as the Bay of Bengal^5^ and the West Africa^6^. Recently, GPS-derived VCD has been proved to be linearly correlated to the TWS variations^7, 8^ and has been used for hydrological drought monitoring in the western United States^9–12^. Moreover, GPS-derived VCD has been found to be not only linearly correlated to TWS variations but also to precipitation variations at the annual period in the Horn of Africa^13^. Furthermore, GPS-derived PWV has been used to study the monsoon cycle in Africa^14^. However, GPS-derived PWV and VCD have not been used jointly to investigate the monsoon cycle or to characterize drought.

Yunnan is situated in southwest China, between the Bay of Bengal and the South China Sea. It is a typical monsoon climate region that is characterized by clear wet and dry seasons, thus prone to suffer from drought and flood disasters. In recent years, several devastating droughts over this region have caused serious economic losses. The extreme drought during winter/spring 2010, which is considered to be the most severe in over a century, affected 8.1 million people and resulted in US$2.5-billion worth of crop damage^15^. It has been demonstrated that droughts in Yunnan are closely associated with monsoon anomalies^16^. Therefore, quantifying monsoon variations is crucial for drought monitoring in Yunnan, which is very important to its socio-economy and agricultural sustainable developments.

Every year, the monsoon brings Yunnan abundant water vapor which falls as precipitation. Precipitation changes TWS and the earth crust deflects in response. If the monsoon is delayed or weak, the water vapor and precipitation will be inadequate which may result in meteorological drought. Inadequate precipitation reduces the replenishment of TWS which could trigger hydrological drought^1^. Precipitation and GRACE-derived TWS have been used to monitor the drought over this region^17^. Furthermore, GPS can observe monsoonal PWV and VCD due to hydrological loading variations, which may provide a good opportunity to extend our knowledge of the monsoon cycle and the characteristics of drought over this region.

This paper aims to investigate whether the annual signal of GPS-derived VCD is correlated to the annual signals of GRACE-derived Equivalent Water Height (EWH) and meteorological precipitation, and, more interestingly, whether the annual signals of VCD and PWV, two types of most widely used GPS observations, are correlated. More importantly, we try to develop the potential of GPS-derived VCD and PWV to help understand the monsoon pattern and characteristics of drought over this region.

Here we use GPS and GRACE time series of about four to eight years to obtain monthly time series of PWV, EWH and VCD in Yunnan. Then annual signals of GPS-derived VCD are compared to those of PWV, and EWH as well as the precipitation from China Meteorological Administration. Moreover, empirical annual amplitude and phase relationships between GPS-derived VCD with PWV, GRACE-derived EWH and meteorological precipitation in Yunnan are obtained. Finally, we test the efficiency of GPS-derived PWV and VCD to monitor drought over this region by comparing their anomalies with those of precipitation and GRACE-derived EWH.

## Data and Methods

**Figure 1:**
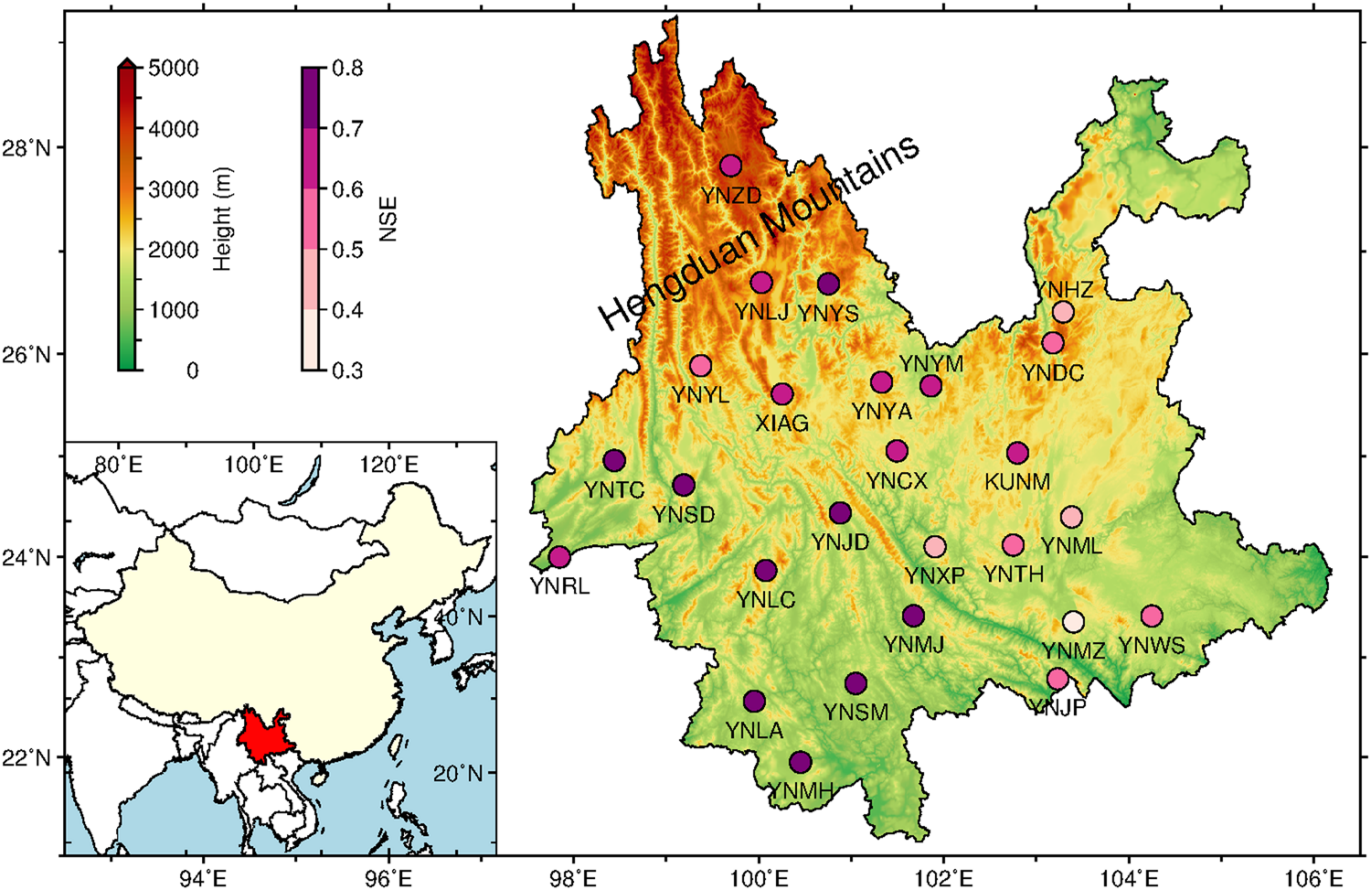
Location map of the 26 Continuous GPS stations in Yunnan, China, and their Nash-Sutcliffe efficiency (NSE) when their GPS-derived VCD are compared with GRACE-derived VCD. This figure is generated by using the Generic Mapping Tools (Version 5.3.1)^26^.

We analyzed 26 Crustal Movement Observation Network of China (CMONOC) GPS stations located in Yunnan by using the GAMIT/GLOBK (Ver. 10.5) software^18^. Most of the stations were observed from 2011.0 to 2015.0, except for the observations of Kunming (KUNM) and Xiaguan (XIAG), which began at 2007.0. The locations of all the stations are shown in Fig. 1. The observation durations of all the stations are listed in Table S1. Daily solutions were generated by estimating the station coordinates, zenith wet delays (ZWD) (every two hour), atmospheric gradients (two pairs per day in N/S and E/W), orbital parameters and other parameters together. Tropospheric delays were corrected with the Vienna Mapping Function 1 (VMF1)^19^ and a priori Zenith Hydrostatic Delay (ZHD) from the European Centre for Medium-Range Weather Forecasts (ECMWF)^20^. The effects of high order ionospheric delay were corrected with the International Geomagnetic Reference Field 11 (IGRF11)^21^ and ionospheric data from the Centre for Orbit Determination in Europe (CODE)^22^. Elevation cutoff angle was set to 10°. The weights of the observations were determined by their elevation angles and post-fit phase residuals. Ocean tide loading was modeled by using the FES2004 model^23^. Meanwhile, we reprocessed observations from 73 globally evenly-distributed GPS stations with the same strategy as that used for the regional network. These stations were divided into two interwoven global subnetworks for efficiency. We then combined the daily loosely constrained solutions of the regional and global networks and transformed them into the IGb08 reference frame^24^ by estimating three translation and three rotation parameters. For hydrological studies, we removed the effects of atmospheric loading from the GPS-derived VCD series of each station by using data provided by the GGFC (van Dam, T., 2010). Vertical annual amplitudes caused by non-tidal oceanic loading are within 1 mm for most inland regions^25^ and thus its contribution was not corrected in the GPS-derived VCD series of this paper.

We aggregated GPS-derived zenith wet delays into daily averages and then converted them to PWV^27^. The weighted mean temperature (Tm) was calculated by using the GTm-III model^28^, in which the annual, semi-annual and diurnal periods were considered. Then, we calculated the monthly sum PWV and detrended monthly mean GPS-derived VCD at each station to correspond to the data binning of precipitation and GRACE, respectively. The linear trend of GPS-derived VCD time series of each station is assumed to be caused by tectonic motion and has been removed. It is difficult to assess whether this assumption is accurate or not and we will address this in the Discussion section.

Precipitation Data from 2007.0 to 2015.0 were derived from the Monthly Surface Precipitation 0.5° × 0.5° Grid Dataset of China (Ver. 2.0) provided by the National Meteorological Information Center of China Meteorological Administration (NMIC/CMA) (http://data.cma.cn/). The monthly precipitation time series at each GPS station were estimated by bilinear interpolation.

We used the fifth release of GRACE Level-2 GSM gravity field models (degree and order 96) from the University of Texas Center for Space Research (CSR) with the same time span as the GPS observations. For hydrological studies, contributions from atmospheric and oceanic mass redistribution have been corrected from the GRACE GSM products. To suppress errors of the gravity spherical harmonic coefficients at high degrees, we used the DDK2 filtered solutions (provided at http://icgem.gfz-potsdam.de/ICGEM/), which is approximately equal to a Gaussian filter with a 340 km smoothing radius^29^. We replaced the C_20_ terms with the estimations from Satellite Laser Ranging observations^30^ (provided at ftp://podaac.jpl.nasa.gov). To be consistent with the GPS-derived VCD, we added back the estimations of degree-1 terms provided by Swenson *et al*.^31^. Then, the mean of the gravity spherical harmonics coefficients was subtracted to estimate the EWH and the VCD due to water mass loading variations at each station with load Love numbers from Farrell^32^. It is worth noting that positive deviation of GPS-derived and GRACE-derived VCD means downward movement in this paper to facilitate comparison with EWH.

**Figure 2:**
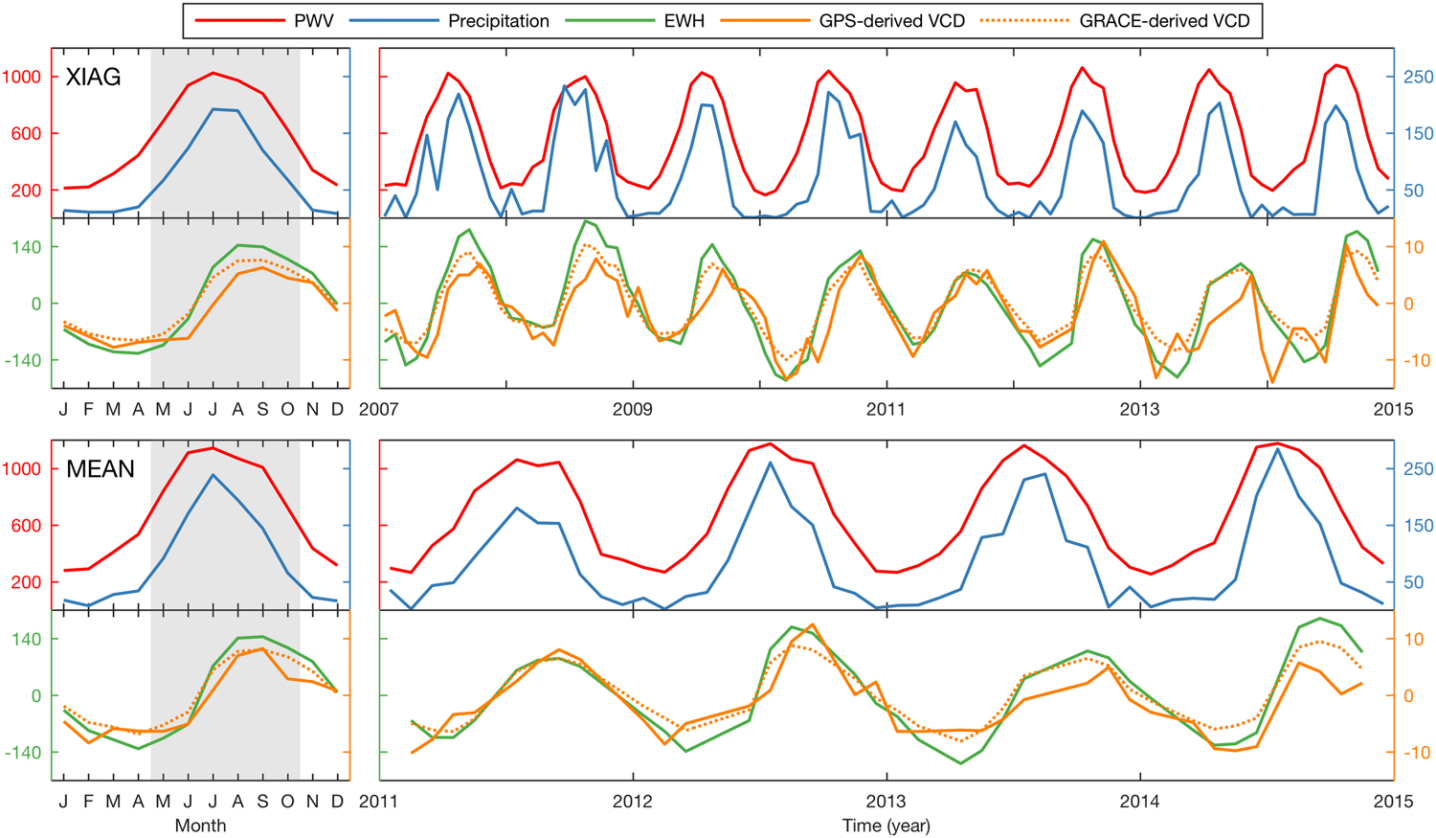
Mean annual signals (left panels) and monthly time series (right panels) of PWV, precipitation, EWH, GPS-derived and GRACE-derived VCD at XIAG (upper panels) station and for the mean time series of 25 stations from 2011.0 to 2015.0 (MEAN, lower panels). To facilitate comparison with EWH, positive deviation of VCD means downward movement. All of these quantities are in millimeters. The gray rectangles indicate the monsoon season.

Thus, we obtained time series of the GPS-derived VCD and PWV, precipitation, and GRACE-derived VCD and EWH at each station. For each quantity, we also obtained the mean time series of 25 stations from 2011.0 to 2015.0 and named it as MEAN in this paper (lower panels of Fig. 2) to represent the average variations of the whole region. For the mean time series, the KUMN station is excluded because its data was missing starting in 2013.1.

GPS-derived VCD is influenced by both water mass loading variations and poroelastic adjustments, whereas GRACE-derived VCD is only influenced by water mass loading variations. Therefore, poroelastic adjustments might damage the consistency between GPS-derived and GRACE-derived VCD, thus limiting the usefulness of GPS-derived VCD in hydrological studies^11, 33^. We adopt the Nash-Sutcliffe efficiency (NSE), a conventional metric in hydrology, to evaluate the consistency between GPS-derived and GRACE-derived VCD time series^34, 35^. NSE is calculated by

1 $${\rm{NSE}}=1-\frac{{\sum }_{i=1}^{n}{({h}_{i}^{GPS}-{h}_{i}^{GRACE})}^{2}}{{\sum }_{i=1}^{n}{({h}_{i}^{GPS}-{\bar{h}}^{GPS})}^{2}}$$

where ${h}_{i}^{GPS}$ and ${h}_{i}^{GRACE}$ are the time series of GPS-derived and GRACE-derived VCD estimations, and ${\bar{h}}^{GPS}$ is the mean value of GPS-derived VCD estimations.

Figure 1 shows 26 GPS stations’ NSE when their GPS-derived VCD are compared with GRACE-derived VCD. The NSEs are larger than 0.50 for most of the stations with an average of about 0.63, indicating that the consistency between GPS-derived and GRACE-derived VCD is very good and GPS-derived VCD is a proper proxy for monitoring the TWS variation in Yunnan.

Inspection of Fig. 2 shows that the time series of all quantities show significant annual variations. To analyse the annual variations, we calculated the mean annual signal of each quantity and each station by stacking its multiyear monthly time series into one year, in which a weighted mean is used for GPS-derived VCD and mean values for other quantities. To make a quantitative analysis for annual variations, we modeled the variations of all the time series as

2 $$f(t)=\sum _{i=1}^{2}{A}_{i}\,\sin ({\omega }_{i}t+{\phi }_{i})$$

where *A*_1_ and φ_1_ are the amplitude and phase of annual signal, and *A*_2_ and φ_2_ are those of semiannual signal, respectively. Then we converted φ_1_ into the day of the year when an annual signal reaches its maximum and use it as the annual phase in this paper, which makes the interpretation more direct^36^. For interannual variation analysis, we calculated each station’s monthly anomalies of these quantities by subtracting their own mean annual signals.

## Results and Analyses

### Annual Variation

**Figure 3:**
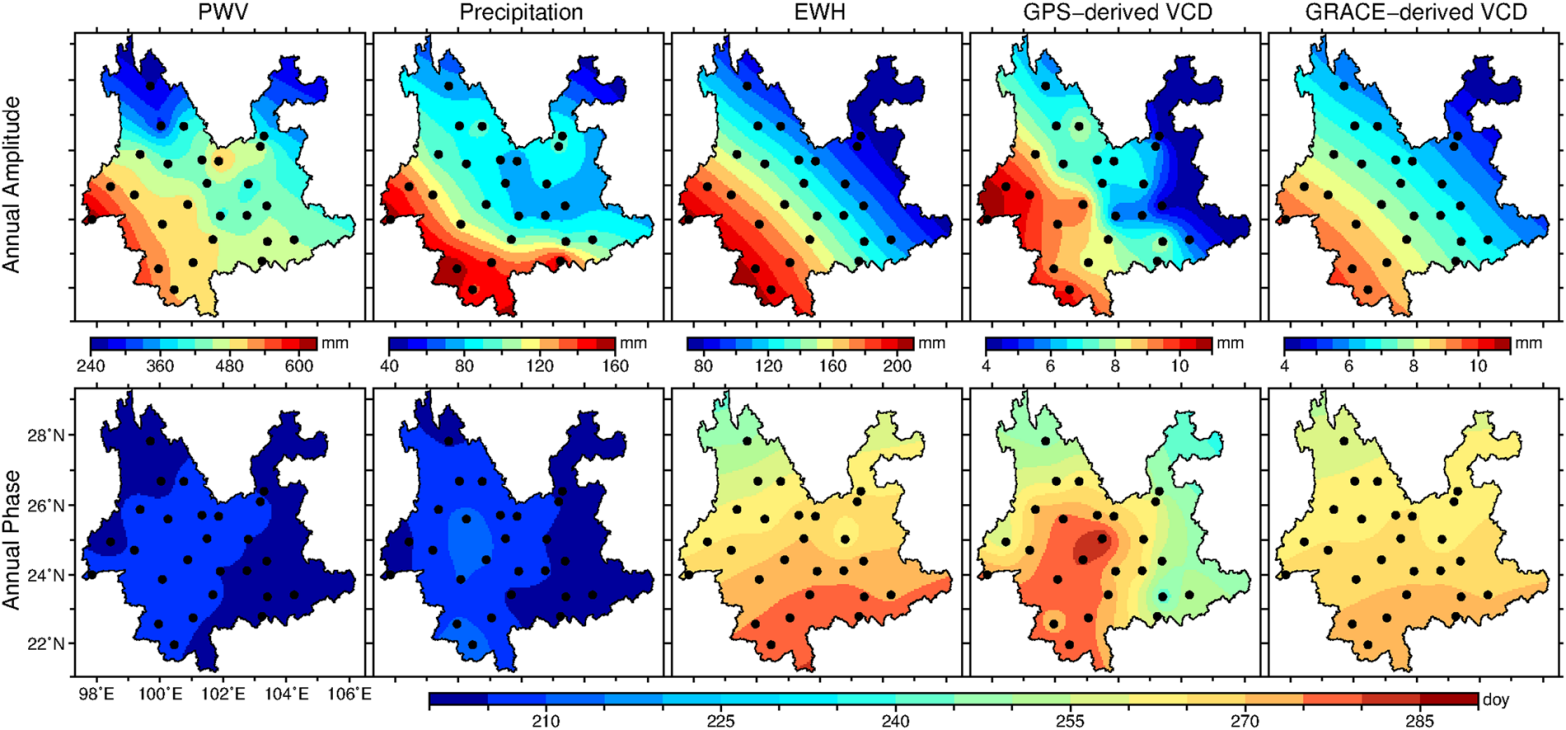
Maps of annual amplitude (upper panel, unit: mm) and phase (lower panel, unit: day of year) of PWV, precipitation, EWH, GPS-derived and GRACE-derived VCD. The annual phase is the day of the year (doy) when an annual signal reaching its maximum. This figure is generated by using the Generic Mapping Tools (Version 5.3.1)^26^.

Figure 3 shows the spatial distribution of annual amplitudes and phases for PWV, precipitation, EWH, GPS-derived and GRACE-derived VCD. The discrete point values at each station are interpolated into grids with adjustable tension continuous curvature and a tension factor of 0.25 and plotted by using the Generic Mapping Tools (Version 5.3.1)^26^. As can be seen from the upper panels of Fig. 3, annual amplitudes of all quantities show a similar spatial variation pattern and increase from northeast to southwest. Southwest Yunnan is closer to the Bay of Bengal which is the water vapor source of the main monsoon. Therefore, the spatial variations of these quantities’ annual amplitude indicate a series of positive correlations that larger PWV’s amplitude corresponds to larger precipitation’s amplitude; larger precipitation’s amplitude corresponds to larger EWH’s amplitude, and larger EWH’s amplitude corresponds to larger GPS-derived VCD’s amplitude. Thus, we estimate the correlation between the annual amplitudes of GPS-derived VCD and the other four quantities in Yunnan.

**Figure 4:**
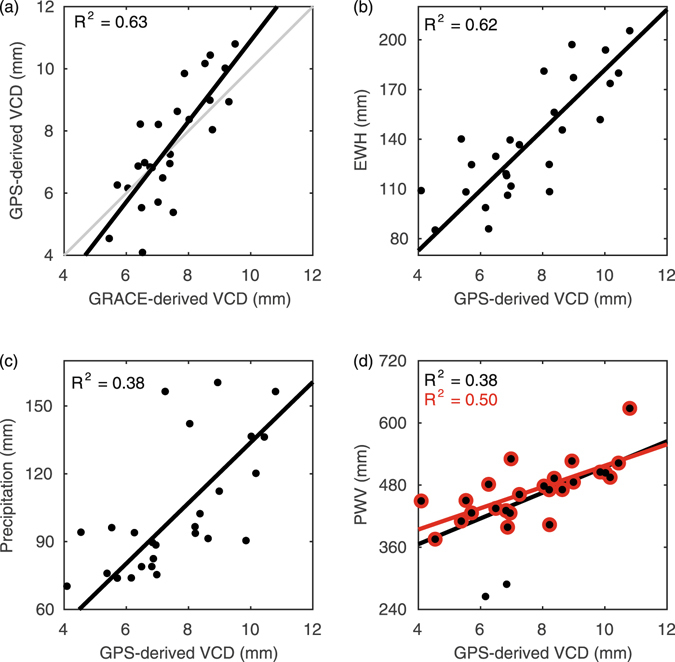
Empirical relations of the annual amplitude between GPS-derived VCD and other quantities. (**a**) for GRACE-derived VCD, (**b**) for EWH, (**c**) for precipitation, and (**d**) for PWV. The red line in (**d**) is the linear fitting line without two abnormal stations, Lijiang (YNLJ) and Shangri-La (YNZD).

Figure 4a shows that the annual amplitude of GPS-derived VCD (*A*_GPS_VCD_) and GRACE-derived VCD (*A*_GRACE_VCD_) are linearly correlated with *A*_GPS_VCD_ = 1.3 *A*_GRACE_VCD_–2.0 (R^2^ = 0.63), which is close to the ideal case that they are equal. Moreover, the annual amplitudes of GPS-derived VCD are expected to be linearly correlated to that of GRACE-derived EWH (*A*_GRACE_RWH_) and meteorological precipitation (*A*_prec_)^13^ as shown in Fig. 4b and 4c. Thus, we can obtain the empirical relations: *A*_GPS_VCD_ = 18.1 *A*_GRACE_EWH_ (R^2^ = 0.62) and *A*_GPS_VCD_ = 13.3 *A*_prec_ (R^2^ = 0.38). These results will be compared with results from the Horn of Africa^13^ in the Discussion section.

Figure 4d shows that annual amplitudes of GPS-derived VCD and PWV (*A*_GPS_PWV_) are also correlated. But Lijiang (YNLJ) and Shangri-La (YNZD) stations’ PWV are identified as outliers for the linear fitting. GPS-derived PWV is an *in-situ* measurement of PWV observed from the altitude of a GPS station, while GPS-derived VCD is mainly sensitive to mass loadings with a horizontal scale of ~100 km^7^. YNLJ and YNZD stations are located in the Hengduan Mountains with huge terrain fluctuations (Fig. 1). That is to say, the annual amplitudes of their GPS-derived PWV cannot reflect the average value of their surrounding areas correctly, whereas the other stations are representative. With YNLJ and YNZD stations excluded, the empirical relation turns from *A*_GPS_VCD_=24.8 *A*_GPS_PWV_ + 266.7 (R^2^ = 0.38) to *A*_GPS_VCD_ = 20.5 *A*_GPS_PWV_ + 312.0 (R^2^ = 0.50).

The lower panel of Fig. 3 illustrates spatial distribution of the annual phases of the PWV, precipitation, EWH, and GPS-derived and GRACE-derived VCD, respectively. Their mean values and standard deviations are listed in Table 1. The annual phases of GPS-derived VCD show the largest spatial variations with a standard deviation of 12 days and a mean value of the 266th day of year while those of PWV show the lowest spatial variations with a standard deviation of 2 days with a mean value of the 205th day of year. Table 1 also shows the statistics of annual phase differences for the PWV, precipitation, EWH and GRACE-derived VCD with respect to GPS-derived VCD. We can find that annual signals of GPS-derived VCD are almost totally in phase with GRACE-derived VCD and EWH. Moreover, the annual signals of PWV and precipitation are also almost totally in phase and their annual phases are about two months earlier than those of EWH and GPS-derived VCD.

**Table 1:** Statistics of annual phase of PWV, precipitation, EWH, and GPS-derived and GRACE-derived VCD and their difference with respect to GPS-derived VCD.

	Annual Phase		Annual Phase difference w.r.t GPS-derived VCD	
	Mean	STD	Mean	STD
PWV	205	2	−61	10
Precipitation	207	4	−60	10
EWH	269	7	2	13
GPS-derived VCD	266	12	—	—
GRACE-derived VCD	266	4	0	12

All these quantities are in days. The annual phase is the day of year when an annual signal reaches its maximum.

To analysis the annual variations of these quantities and their relationships in the study region, water balance equation is introduced as follows^37^:

3 $$\frac{dTWS}{dt}=P+{R}_{{\rm{in}}}-{R}_{{\rm{out}}}-ET$$

where $\frac{dTWS}{dt}$, *P*, *R*_in_, *R*_out_ and *ET* are the monthly total water storage variations, monthly precipitation, inflow, outflow and evapotranspiration for this region, respectively. The sum of *P* and *R*_in_ is the input of TWS and the sum of *R*_out_ and *ET* is the output of TWS of this region.

Monthly TWS variations and precipitation in Yunnan are reflected by the average EWH variations and precipitation at the GPS stations investigated in this paper. According to Equation (3), the phase relationships shown in Table 1 and the left lower panel of Fig. 2, the influences of the monsoon on the water balance of Yunnan can be divided into four phases as follows:

1. Every year, PWV and precipitation increase slowly from February to April while EWH keeps decreasing, indicating that TWS input is still lower than its output though precipitation increases during this period.
2. With the monsoon onset in May, PWV and precipitation increase swiftly and reach their maximums in July with the strongest monsoon intensity. Meanwhile, EWH keeps accumulating. These results indicate that the TWS input starts to be larger than its output with a significant precipitation increase during this period.
3. Then, PWV and precipitation decrease gradually from July to September but still with relatively large values while EWH still keeps accumulating during this time, indicating that the TWS input is still larger than its output although precipitation decreases during this period.
4. With the monsoon retreat in October, PWV and precipitation decrease swiftly and reach their minimums in February of the next year. Meanwhile, EWH reaches its peak in September and then keeps decreasing. These results indicate that the TWS input starts to be lower than its output with a significant precipitation decrease during this period.

Thus, an annual monsoon cycle is completed and the two-month phase lag between the annual signals of EWH and precipitation is explained accordingly. Figure 2 shows that annual variations of PWV and precipitation are generally consistent as PWV is the source of precipitation. Moreover, Figure 2 also shows that annual variations of GPS-derived VCD and GRACE-derived EWH are generally consistent, indicating an elastic response of the crust to the hydrological mass loading. Therefore, the two-month phase lag between the annual signals of GPS-derived VCD and PWV is also explained according to these geophysical processes.

### Interannual Variation

The onset and duration of the monsoon and its intensity vary from year to year and this could trigger droughts and floods. In this section, we analyze the interannual variations of these quantities to test whether GPS-derived VCD and PWV have the capability to capture the signals of droughts.

**Figure 5:**
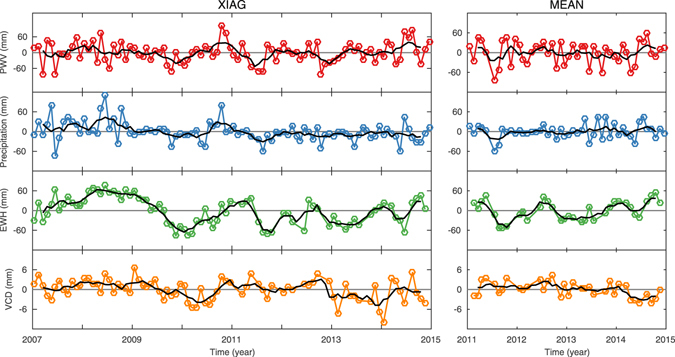
Monthly anomalies of PWV (*red curves*), precipitation (*blue curves*), EWH (*green curves*) and GPS-derived VCD (*orange curves*) at XIAG station and for MEAN, respectively. Their five-month moving average time series (black curves) are also shown.

The left panel of Fig. 5 illustrates the PWV, precipitation, EWH and GPS-derived VCD anomaly time series for XIAG station as its observing time is the longest (about 8 years). Smoother variations of these anomaly series are obtained with a five-month moving average filter and shown as black curves in Fig. 5. Significant precipitation deficits occur in June 2007, October 2009, May and June 2010, August 2011, October 2012, June 2013 and May 2014. For many other months, precipitation anomalies show slight oscillations. However, significant EWH deficits occur in similar months but with longer durations, such as in winter/spring 2010, winter/spring 2012 and during winter/spring 2013. The inconsistency in their duration may be caused by the fact that in XIAG’s monsoon season (from May to October), precipitation accounts for 91% of the entire annual precipitation, and thus, a short but large precipitation deficit in the monsoon season could lead to long-lasting EWH deficit until effective replenishment occurs. In particular, the significant precipitation deficits at the end of the monsoon season are likely to trigger a long-lasting EWH deficit in the subsequent non-monsoon season. This might be an interpretation for the above three winter/spring EWH deficit periods. Therefore, these periods are considered to be drought periods which are consistent with *Qiu* [2010]^15^, *Long et al*.^37^, and *Tang et al*.^17^.

**Figure 6:**
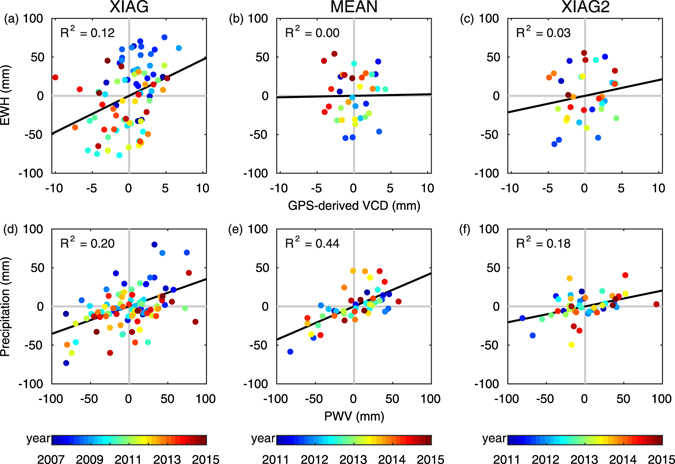
Scatterplots of GPS-derived VCD anomalies versus EWH anomalies and PWV anomalies versus precipitation anomalies at XIAG station and for MEAN. Anomalies obtained from the time series of XIAG station from 2011.0 to 2015.0 is termed as XIAG2 station in this paper.

Inspection of the left panel of Fig. 5 shows that GPS-derived VCD anomalies also stay negative during the winter/spring 2010 and winter/spring 2013 drought periods, similar to EWH anomalies. However, abnormal GPS-derived VCD anomalies can be seen in December 2013 and January 2014 for unknown reasons and need further investigation. Furthermore, the uncertainty of the monthly GPS-derived VCD is about 1 mm, smaller than the anomalies in the drought months, suggesting that the anomaly signals are the instantaneous crustal response due to hydrological loading anomalies rather than noise. Figure 6a also shows that XIAG’s GPS-derived VCD anomalies are generally proportional to its GRACE-derived EWH. Therefore, negative GPS-derived VCD anomalies may serve as an indicator of drought in Yunnan.

Comparison of Fig. 6a and 6d shows that the consistency between XIAG’s PWV anomalies and precipitation anomalies is better than that between the GPS-derived VCD anomalies and GRACE-derived EWH anomalies. PWV shows large deficits, especially for the months with large precipitation deficits, indicating that PWV anomalies are capable of detecting the drought signals and can be seen as a supplementary means to help to diagnose the drought that caused by the monsoon anomaly.

In addition, we have also calculated the anomalies for MEAN (right panel of Fig. 5). Its PWV and precipitation anomalies show a good consistency. Both of them show large deficits in the summer of 2011 and the spring of 2014. Moreover, we find that PWV and precipitation anomalies share the same signs for 74.5% months. That is to say, even with a relatively short period of records, PWV and precipitation anomalies can also show relatively high correlations (Fig. 6e). However, the consistency between their GPS-derived VCD and GRACE-derived EWH are rather poor which share the same signs for 54.3% months (Fig. 6b). Although regional TWS deficit during winter/spring 2012 and winter/spring 2013 can be identified by the GRACE-derived EWH anomalies, the signals identified by GPS-derived VCD are not so conspicuous. We suspect the reason is that four years of GPS-derived VCD time series is too short to obtain a real linear tectonic velocity, and this could result in its unreal and inaccuracy anomalies. To verify this assumption, we use the time series of XIAG from 2011.0 to 2015.0 to form a new station named XIAG2. We calculate its anomalies of all quantities with the same methods as other stations that described in Section 2. Comparing Fig. 6a and 6c, we can find that the consistency of GPS-derived VCD and EWH are rather poor for four years’ time series but much better for eight years’ time series. Thus, we can infer that GPS-derived VCD also have the potential to monitor drought but may need longer observations.

## Discussion

With respect to hydrometeorology, PWV, precipitation and TWS are critical components for the hydrological and monsoon cycle. In Yunnan, all of the time series of PWV, precipitation, EWH and GPS-derived VCD show typical annual signals governed by the monsoon cycle. A phase lag of approximate two months between the annual signals of EWH and precipitation in Yunnan is found, which is also found in other monsoonal regions, such as India^38^ and the Horn of Africa^13^. Moreover, with the GPS products introduced in this paper, we found that the annual phases of PWV and precipitation are consistent and those of GPS-derived VCD and GRACE-derived EWH are also consistent. These results are conducive to understand the mechanisms of monsoon and hydrological cycle in Yunnan and may serve as a basis for characterizing the interannual variabilities of monsoon.

With respect to drought monitoring, the GPS-derived VCD anomalies should contain purely hydrological signals with other signals removed. We find that most of the stations show a linear trend from −3 to 2 mm yr^−1^ in the vertical, which could be due to long-term changes in TWS as well as tectonic effects. Though we removed the linear trend of the GPS-derived VCD time series as stated in the Method section, the effects of long-term changes in TWS is discarded at the same time which could lead to the wrong interpretation of drought. Thus, we also calculate the GPS-derived VCD anomalies without removing the linear trend and compare them with VCD anomalies with the linear trend removed. The results show that these stations’ GPS-derived VCD and EWH anomalies share the same signs at 50.2% and 53.3% months on average, respectively. We therefore recommend using GPS-derived VCD anomalies with the linear trend removed to be used for drought monitoring in Yunnan. This approach is also adopted to monitor drought in the High Plains of the United States^11^. Meteorological and hydrological drought are not the same^1^ and they can be identified by precipitation deficit and TWS deficit, respectively. In this paper, we find that PWV and precipitation anomalies of MEAN share the same signs for 74.5% of the months. On the other hand, although the consistency between the anomalies of GPS-derived VCD and EWH is not good for XIAG with four years data, we find that their consistency is better with eight years data. These results indicate that GPS-derived PWV and VCD provide valuable information to help us identify and distinguish meteorological and hydrological drought in support of near real-time data.

With respect to the solid Earth, the empirical scaling factors of annual amplitudes between GPS-derived VCD with GRACE-derived EWH and meteorological precipitation we estimated for Yunnan are 18.1 and 13.3, respectively. However, the values for the Horn of Africa are 8.6 and 13.8, respectively^13^. It should be noted that the effects of atmospheric loading have been removed from our GPS-derived VCD while kept unchanged in Birhanu and Bendick^13^. To test whether atmospheric loading might result in the differences, we also estimate the empirical scaling relations by using the GPS-derived VCD without applying atmospheric loading corrections as shown in Table S2. However, the results are 17.0 and 12.4 for GPS-derived VCD with EWH and precipitation, suggesting that atmospheric loading is not the main cause of the differences. This might be related to the differences in spatial extent of seasonal hydrological loading and the differences of crust and mantle properties^11^ between Yunnan and the Horn of Africa. Testing these hypotheses would require data collection and analysis beyond the scope of the current paper. Furthermore, by comparing the empirical relationships, we may infer that the variations of EWH in Yunnan are about twice of those in the Horn of Africa with the same variations of precipitation, which may be related to the difference in water storage capability between this two areas. This hypothesis will be tested in our future work.

To evaluate the consistency between GPS-derived and GRACE-derived VCD in Yunnan and compare the results with other regions, we calculated NSE and WRMS Reduction^39^ as shown in Table S3. The results show that consistency in Yunnan is much higher than that in Europe^35^ and globally^36^, and close to that in Nepal^40^ and the Amazon basin^35^ with the largest annual TWS variations in the world. We attribute this to the almost purely elastic response of the crust due to hydrological loading in Yunnan as well as the homogeneous reprocessing of GPS data with the latest strategies. However, there are some other effects that could weaken the consistency, such as thermal expansion, the aliased tidal residuals, the draconitic period and the difference in spatial resolution between the GPS and GRACE data. Thermal expansion of GPS monuments and the nearby bedrock can induce vertical displacements that cannot be seen in GRACE. The annual amplitude of the thermal expansion at Kunming (KUNM) and Xiaguan (XIAG) are no more than 0.4 mm^41, 42^. As other stations’ monument height and material, crust property and temperature variation patterns are similar to KUNM and XIAG, we may speculate that the thermal expansion at these stations are also negligible, as their typical annual amplitudes are 6–10 mm. In addition, magnitudes of the effect of aliased sub-daily and draconitic signals in GPS height time series are typically within 1 mm^43^ and 0.03 mm^12^, respectively, which are also much smaller than the amplitude of GPS-derived VCD due to hydrological loading here. GPS-derived VCD is mainly sensitive to the loads within ~100 km^7^, while the spatial resolution of GRACE is about 300 km, which means they reflect loading variations at different spatial scales. Moreover, the filter in GRACE data processing makes the results smoother in space^35^. All of these factors contribute to the remaining inconsistency between GPS-derived and GRACE-derived VCD.

## Conclusion

This is the first study combining PWV and VCD, two types of common GPS products, to analyze the monsoon cycle and drought associated with monsoon anomalies. A case study of 26 Continuous GPS stations located in Yunnan, a typical monsoon region, demonstrates that all of the time series of precipitation, EWH and GPS-derived PWV and VCD show typical annual signals that are associated with the monsoon cycle. Significant phase relationships can be seen from their annual signals. For instance, GPS-derived VCD (positive means downward movement) is in phase with EWH and PWV is in phase with precipitation. Our results demonstrate that the annual signals of VCD and PWV show linearly correlated amplitudes (*A*_GPS_VCD_ = 20.5 *A*_GPS_PWV_ + 312.0 (R^2^ = 0.50)) and a stable phase lag of about two months. The annual amplitudes of precipitation and EWH also show similar relations to that of VCD here: *A*_GPS_VCD_ = 13.3 *A*_prec_ (R^2^ = 0.38) and *A*_GPS_VCD_ = 18.1 *A*_GRACE_EWH_ (R^2^ = 0.62). These relationships indicate that GPS supports a more comprehensive understanding of the monsoon pattern and its influences over not only Yunnan but also other monsoon regions, such as South Asia, West Africa and South America.

As for drought monitoring, we find that for the mean time series of 25 stations from 2011.0 to 2015.0 termed as MEAN in this paper, its PWV and precipitation anomalies share the same signs for 74.5% months, while its GPS-derived VCD and EWH anomalies share the same signs for 54.3% months. That is to say GPS-derived PWV and VCD are capable of providing drought-related information, which may serve as a new means for drought monitoring and analysis. The GPS technique, with the advantages of near-real time capability and high temporal and spatial resolution, has a great potential not only for the solid Earth studies but also for hydrometeorology.

## Electronic supplementary material

Supporting information
