# Supplementary material for: Annual variations of monsoon and drought detected by GPS: A case study in Yunnan, China
Source: Sci Rep. 2017 Jul 19;7:5874. doi: 10.1038/s41598-017-06095-1 (PMC5517602; doi:10.1038/s41598-017-06095-1)
Supplement: Supplementary file 1 — Supporting information [file 41598_2017_6095_MOESM1_ESM.pdf]

Supporting Information

Annual variations of monsoon and drought detected by GPS：A case study in Yunnan, China

Weiping Jiang[1 *], Peng Yuan[1,3], Hua Chen[2], Jianqing Cai[3], Zhao Li[4], Nengfang Chao[5] and Nico Sneeuw[3]

1 GNSS Research Center, Wuhan University, Wuhan 430079, China

2 School of Geodesy and Geomatics, Wuhan University, Wuhan 430079, China

3 Institute of Geodesy, University of Stuttgart, Stuttgart 70174, Germany

4 Faculté des Sciences, de la Technologie et de la Communication, University of Luxembourg, 6, rue Richard Coudenhove-Kalergi, L-1359 Luxembourg City, Luxembourg

5 MOE Key Laboratory of Fundamental Physical Quantities Measurement, School of Physics, Huazhong University of Science and Technology, Wuhan 430074, China

* Corresponding author: wpjiang@whu.edu.cn

Table S1. GPS station locations and observation epochs used in this study.

Site	Longitude (°)	Latitude (°)	Height (m)	Duration (yr)
KUNM	102.8	25.0	1986.2	2007.0 – 2013.1
XIAG	100.3	25.6	1974.3	2007.0 – 2015.0
YNCX	101.5	25.1	1785.4	2011.0 – 2015.0
YNDC	103.2	26.1	1297.6	2011.0 – 2015.0
YNHZ	103.3	26.4	2263.9	2011.0 – 2015.0
YNJD	100.9	24.4	1244.6	2011.0 – 2015.0
YNJP	103.2	22.8	1250.1	2011.0 – 2015.0
YNLA	100.0	22.6	1107.9	2011.0 – 2015.0
YNLC	100.1	23.9	1559.5	2011.0 – 2015.0
YNLJ	100.0	26.7	3195.8	2011.0 – 2015.0
YNMH	100.5	22.0	1166.3	2011.0 – 2014.6
YNMJ	101.7	23.4	1281.7	2011.0 – 2015.0
YNML	103.4	24.4	1520.8	2011.0 – 2015.0
YNMZ	103.4	23.4	1275.3	2011.0 – 2015.0
YNRL	97.9	24.0	723.3	2011.0 – 2015.0
YNSD	99.2	24.7	1478.8	2011.0 – 2015.0
YNSM	101.1	22.7	1445.4	2011.0 – 2015.0
YNTC	98.4	25.0	1470.1	2011.0 – 2015.0
YNTH	102.8	24.1	1778.2	2011.0 – 2015.0
YNWS	104.3	23.4	1451.6	2011.0 – 2015.0
YNXP	101.9	24.1	1978.0	2011.0 – 2015.0
YNYA	101.3	25.7	1846.2	2011.0 – 2015.0
YNYL	99.4	25.9	1696.3	2011.0 – 2015.0
YNYM	101.9	25.7	1077.8	2011.0 – 2015.0
YNYS	100.8	26.7	2143.9	2011.0 – 2015.0
YNZD	99.7	27.8	3296.9	2011.0 – 2015.0

Table S2. Comparison of the empirical scaling factors of annual amplitudes between GPS-derived VCD with EWH and precipitation.

VCD	EWH		Precipitation	
	scaling factor	R^2	scaling factor	R^2
with ATML correction, Yunnan, present paper	18.1	0.62	13.3	0.38
without ATML correction, Yunnan, present paper	17.0	0.68	12.4	0.32
without ATML correction, the Horn of Africa, Birhanu and Bendick (2015)	8.6	0.76	13.8	0.64

Table S3. Comparison of the consistency between GPS-derived and GRACE-derived VCD.

Region	WRMS Reduction (%)	NSE
Yunnan, China, present paper	44	0.63
Europe, Chen (2015)	29	0.47
Amazon, Chen (2015)	46	0.64
Nepal, Fu and Freymueller (2012)	45	-
Global, Tesmer et al. (2011)	12	-
